# Supplementary material for: Effect of COVID-19 pandemic on missed medical appointment among adults with chronic disease conditions in Northwest Ethiopia
Source: PLoS One. 2022 Oct 4;17(10):e0274190. doi: 10.1371/journal.pone.0274190 (PMC9531804; doi:10.1371/journal.pone.0274190)
Supplement: S1 Text — (DOCX) [file pone.0274190.s001.docx]

**English version questionnaire**

**Section I: Socio-Demographic Characteristics**

| S/no | Questions | Response categories |
| --- | --- | --- |
|  | Medical Registration Number (MRN) | ______________ |
|  | Zone______________, Woreda ______________ | Kebele___________ |
|  | Age | ----------- years |
|  | Sex | 1. Male 2. Female |
|  | Religion | 1. Orthodox 2. Muslim 3. Protestant 4. Catholic 5. Others |
|  | Residence | 1. Urban 2. Rural |
|  | Marital status | 1. Single 2. Married 3. Divorced 4. Separated 5. Widowed |
|  | Occupation | 1. Government employee 2. Private employee 3. Farmer 4. Merchant 5. House wife 6. Student 7. Unemployed 8. Other, specify_____ |
|  | Level of education | 1. Unable to read & write 2. Only read and write 3. Primary education 4. Secondary education 5. Diploma 6. Degree and above |
|  | Monthly income | ---------Birr |
|  | Living arrangement in the family (multiple choice is possible) | 1. Head of the family 2. Mother 3. Father 4. Daughter 5. Son 6. Relative 7. Other, specify______ |
|  | Payment method for health service | 1. Health insurance 2. Poverty card 3. Out of pocket |
|  | Distance from your home to this hospital (in KM)? | ______________ |

**Section II: Clinical Factors**

| S/no | Questions | Response category | | |
| --- | --- | --- | --- | --- |
|  | Type of main diagnosis? | 1. HIV/AIDS 2. Diabetes 3. Hypertension 4. COPD 5. Asthma 6. chronic liver diseases 7. CKD 8. cardiac illnesses 9. Cancer | | |
|  | Date of main diagnosis | (dd/mm/yy)_____/____/_____ | | |
|  | Duration on follow up (months) | ____________ | | |
|  | Have you missed any appointment in the last one year before COVID-19 pandemic (March 13, 2019 to 2020) | 1. Yes 2. No 3. I don’t remember | | |
|  | If yes to Q19, how many visits have you missed? (check **chart**) | _______________ | | |
|  | Treatment outcome before March 13, 2020 (**check** **chart**) | 1. Well controlled/stable 2. Fairly controlled 3. Poorly controlled/deteriorating/progressing | | |
|  | Have you missed any appointment during COVID-19 pandemic (Since march 13, 2020) | 1. Yes 2. No | | |
|  | If yes to Q22, how many visits have you missed? | _______________ | | |
|  | What are the reasons for missed visit? (multiple response) | 1. Fear of COVID-19 infection 2. Health facility locked down 3. Health professionals were not cooperative 4. Transportation problem due to COVID-19 state of emergency 5. Others, specify________ | | |
|  | Have you ever had emergency visit or admission after COVID-19 pandemic? | 1. Yes 2. No | | |
|  | Do you have any comorbidity? | 1. Yes 2. No | | |
|  | If yes to Q26, which comorbidity? (multiple answer is possible) | 1. HIV/AIDS 2. Diabetes 3. Hypertension 4. COPD 5. Asthma 6. Chronic liver diseases 7. CKD 8. Cardiac illnesses 9. Cancer 10. Stroke 11. Epilepsy/seizure 12. SLE/RA 13. IBD 14. Anemia 15. Others, specify | | |
|  | Have you had unusual sudden onset of the following symptoms during COVID-19 pandemic? | 1. Dry cough 2. High grade fever 3. Loss of smell 4. Muscle/joint ache 5. Headache 6. Shortness of breath | | |
|  | Have you been diagnosed with COVID-19 infection? | 1. Yes 2. No | | |
|  | If yes to Q29, where did you received the care? | 1. Home 2. Health facility | | |
|  | If no to Q29, do you perceive you had COVID 19 infection? | 1. Most likely 2. Likely 3. Less likely 4. I do not know | | |
|  | Type of medication | - 1. Oral   2. Injectables   3. Both | | |
|  | How many times in a day do you take medications? | __________ | | |
|  | How many kinds of medication do you take? | __________ | | |
|  | How frequent is your follow up appointment? | 1. Weekly 2. Every two/three weeks 3. Monthly 4. Every three months 5. Every six months or more | | |
|  | Do you have any identified complications of the chronic illness? (verbaly and chart) | 1. Yes 2. No | | |
|  | If yes, specify? | ______________ | | |
|  | Treatment outcome during COVID-19 pandemic | 1 | 2 | 3 |
|  | Visit 1 |  |  |  |
|  | Visit 2 |  |  |  |
|  | Visit 3 |  |  |  |
|  | Visit 4 |  |  |  |
|  | Date of first visit | (dd/mm/yyyy)_____/_____/_____ | | |
|  | Date of last visit | (dd/mm/yyyy)_____/_____/_____ | | |

Note: 1 = Well controlled/stable, 2 = Fairly controlled, 3 = Poorly controlled/deteriorating/progressing

| **Section IV: Assessing adherence using the 8 item Morisky medication adherence level** | | | |
| --- | --- | --- | --- |
|  | Do you sometimes forget to take your pills? | 1. Yes 2. No |  |
|  | Thinking over the past two weeks, were there any days when you did  not take your medicine? | 1. Yes 2. No |  |
|  | Have you ever stopped taking your  medicine without telling your doctor because you felt worse when you took it? | 1. Yes 2. No |  |
|  | When you travel or leave home, do you sometimes  forget to bring along your medicine? | 1. Yes 2. No |  |
|  | Did you take all your medicine yesterday? | 1. Yes 2. No |  |
|  | When you feel like your symptoms are under control, do you sometimes stop taking your medicine? | 1. Yes 2. No |  |
|  | Do you ever feel hassled about  sticking to your treatment plan? | 1. Yes 2. No |  |
|  | How often do you have difficulty remembering to take all your medicine? | 1. Never/rarely 2. Once in a while 3. Sometimes 4. Usually 5. All the time |  |

**Section IV: Social Support (OSLO-3)**

| **S/no** | **Questions and filters** | **Response categories** | **Skip to** |
| --- | --- | --- | --- |
|  | How many people are so close to you that you can count on them if you have great personal problems? | 1. None 2. 1–2 3. 3–5 4. 5+ |  |
|  | How much interest and concern do people show in what you do? | 1. None 2. Little 3. Uncertain 4. Some 5. A lot |  |
|  | How easy is it to get practical help from neighbors if you should need it? | 1. Very difficult 2. Difficult 3. Possible 4. Easy 5. Very easy |  |

**Section V: Behavioral factors**

- 1. **Alcohol consumption (AUDIT)**

| **S/no** | **Question** | **Responses** |
| --- | --- | --- |
|  | How often do you have a drink containing alcohol? | 1. Never [Skip to next section] 2. Monthly or less 3. 2 to 4 times a month 4. 2 to 3 times a week 5. 4 or more times a week |
|  | What type of alcohol do you drink? | 1. Beer 2. Tella/corefie 3. Arekie 4. Tej 5. Wine 6. Wuski |
|  | How much do you drink in one occasion? | _____small size tin tela/corefie  _____large size tin tella/corefie  _____glass (bichere) of tella/corefie  _____bottle beer  _____glass of wine  _____flask Tej  ______Melekya Arekie/vodka |
|  | How often do you have six (female)/eight(male) or more drinks on one occasion? | 1. Never 2. Less than monthly 3. Monthly 4. Weekly 5. Daily or almost daily |
|  | How often during the last year have you found that you were not able to stop drinking once you had started? | 1. Never 2. Less than monthly 3. Monthly 4. Weekly 5. Daily or almost daily |
|  | How often during the last year have you failed to do what was normally expected from you because of drinking? | 1. Never 2. Less than monthly 3. Monthly 4. Weekly 5. Daily or almost daily |
|  | How often during the last year have you needed a first drink in the morning to get yourself going after a heavy drinking session? | 1. Never 2. Less than monthly 3. Monthly 4. Weekly 5. Daily or almost daily |
|  | How often during the last year have you had a feeling of guilt or remorse after drinking? | 1. Never 2. Less than monthly 3. Monthly 4. Weekly 5. Daily or almost daily |
|  | How often during the last year have you been unable to remember what happened the night before because you had been drinking? | 1. Never 2. Less than monthly 3. Monthly 4. Weekly 5. Daily or almost daily |
|  | Have you or someone else been injured as a result of your drinking? | 1. No 2. Yes, but not in the last year 3. Yes, during the last year |
|  | Has a relative or friend or a doctor or another health worker been concerned about your drinking or suggested you cut down? | 1. No 2. Yes, but not in the last year 3. Yes, during the last year |

- 1. **Smoking and khat use**

| **S/no** | **Questions and filters** | **Response categories** | **Skip to** |
| --- | --- | --- | --- |
|  | Have you ever smoked cigarette in your lifetime? | 1. Yes 2. No | If no skip to next section |
|  | If yes to Q63, have you smoked in the past one month? | 1. Yes 2. No |  |
|  | If yes to Q64, how many times did you smoke in the past month? | _________times |  |
|  | How many cigarettes on average do you smoke in a day? | _______cigarette/s |  |
|  | Do you currently smoke or use any other type of tobacco? | 1. Yes 2. No |  |
|  | If yes to Q67, what other type of tobacco do you currently smoke or use? (multiple answer is possible) | 1. Pipe  2.Chewing tobacco  3.Snuff/suret  4. Shisha  5. Gaya  6. if other, specify |  |
|  | Is there anyone who used to smoke in your home? | 1. Yes  2. No |  |
| **Khat chewing** | | | |
|  | Have you ever chewed khat? | 1.Yes  2. No |  |
|  | If yes to Q70, did you chew khat in the past one month? | 1.Yes  2. No |  |
|  | If yes to Q71, how many days did you chew khat during the last month? | __________day(s) |  |

- 1. **Physical activity (GPAQ)**

| **Sr/No** | **Questions** | **Response** | **Code** |
| --- | --- | --- | --- |
|  | Does your work involve vigorous-intensity activity that causes large increases in breathing or heart rate like [carrying or lifting heavy loads, digging or construction work] for at least 10 minutes continuously? | 1. Yes 2. No   If No, go to P 4 | P1 |
|  | In a typical week, on how many days do you do vigorous-intensity activities as part of your work? | Number of days_________ | P2 |
|  | How much time do you spend doing vigorous-intensity activities at work on a typical day? | Hours: minutes ____: ____ | P3 |
|  | Does your work involve moderate-intensity activity, that causes small increases in breathing or heart rate such as brisk walking [or carrying light loads] for at least 10 minutes continuously? | 1. Yes 2. No   If No, go to P 7 | P4 |
|  | In a typical week, on how many days do you do moderate-intensity activities as part of your work? | Number of days__________ | P5 |
|  | How much time do you spend doing moderate-intensity activities at work on a typical day? | Hours: minutes ____: ____ | P6 |
|  | Do you walk or use a bicycle *(pedal cycle)* for least 10 minutes continuous to get to and from places? | 1. Yes 2. No   *If No, go to P 10* | P7 |
|  | In a typical week, on how many days do you walk or bicycle for at least 10 minutes continuously to get to and from places? | Number of days_________ | P8 |
|  | How much time do you spend walking or bicycling for travel on a typical day? | Hours: minutes ____: ____ | P9 |
|  | Do you do any vigorous-intensity sports, fitness or recreational *(leisure)* activities that cause large increases in breathing or heart rate like *[running or football]* for at least 10 minutes continuously? | 1. Yes 2. No   *If No, go to P 13* | P10 |
|  | In a typical week, on how many days do you do vigorous- intensity sports, fitness or recreational *(leisure)* activities? | Number of days________ | P11 |
|  | How much time do you spend doing vigorous-intensity sports, fitness or recreational activities on a typical day? | Hours: minutes ____: ____ | P12 |
|  | Do you do any moderate-intensity sports, fitness or recreational *(leisure)* activities that cause a small increase in breathing or heart rate such as brisk walking*, [cycling, swimming, volleyball]* for at least 10 minutes continuously? | 1. Yes 2. No   *If No, go to P16* | P13 |
|  | In a typical week, on how many days do you do moderate- intensity sports, fitness or recreational *(leisure)* activities? | Number of days_____ | P14 |
|  | How much time do you spend doing moderate-intensity sports, fitness or recreational *(leisure)* activities on a typical day? | Hours: minutes ____: ____ | P15 |
|  | How much time do you usually spend sitting or reclining on a typical day? | Hours: minutes ____: ____ | P16 |

**Section VI: Health Related Quality Life (WHO_HRQOL_BREF)**

|  | How would you rate your quality of life? | 1. Very poor 2. poor 3. Neither poor nor good 4. Good 5. Very good |  |
| --- | --- | --- | --- |
|  | How satisfied are you with your health? | 1. Very dissatisfied 2. Dissatisfied 3. Neither satisfied nor dissatisfied 4. Satisfied 5. Very satisfied |  |
| The following questions ask about **how much** you have experienced certain things in the last two weeks. | | | |
|  | To what extent do you feel that physical pain prevents you from doing what you need to do? | 1. Not at all 2. A little 3. A moderate amount 4. Very much 5. An extreme amount |  |
|  | How much do you need any medical treatment to function in your daily life | 1. Not at all 2. A little 3. A moderate amount 4. Very much 5. Extremely |  |
|  | How much do you enjoy life? | 1. Not at all 2. A little 3. A moderate amount 4. Very much 5. Extremely |  |
|  | To what extent do you feel your life to be meaningful? | 1. Not at all 2. A little 3. A moderate amount 4. Very much 5. Extremely |  |
|  | How well are you able to concentrate? | 1. Not at all 2. A little 3. A moderate amount 4. Very much 5. Extremely |  |
|  | How safe do you feel in your daily life? | 1. Not at all 2. A little 3. A moderate amount 4. Very much 5. Extremely |  |
|  | How healthy is your physical environment? | 1. Not at all 2. A little 3. A moderate amount 4. Very much 5. Extremely |  |
| The following questions ask about **how completely** you experience or were able to do certain things in the last two weeks | | | |
|  | Do you have enough energy for everyday life? | 1. Not at all 2. A little 3. Moderately 4. Mostly 5. Completely |  |
|  | Are you able to accept your bodily appearance? | 1. Not at all 2. A little 3. Moderately 4. Mostly 5. Completely |  |
|  | Have you enough money to meet your needs? | 1. Not at all 2. A little 3. Moderately 4. Mostly 5. Completely |  |
|  | How available to you is the information that you need in your day-to-day life? | 1. Not at all 2. A little 3. Moderately 4. Mostly 5. Completely |  |
|  | To what extent do you have the opportunity for leisure activities? | 1. Not at all 2. A little 3. Moderately 4. Mostly 5. Completely |  |
|  | How well are you able to get around? | 1. Not at all 2. A little 3. Moderately 4. Mostly 5. Completely |  |
| The following questions ask you to say how **good** or **satisfied** you have felt about various aspects of your life over the last two weeks. | | | |
|  | How satisfied are you with your sleep? | 1. Very dissatisfied 2. Dissatisfied 3. Neither dissatisfied nor satisfied 4. Satisfied 5. Very satisfied |  |
|  | How satisfied are you with your ability to perform your daily living activities? | 1. Very dissatisfied 2. Dissatisfied 3. Neither dissatisfied nor satisfied 4. Satisfied 5. Very satisfied |  |
|  | How satisfied are you with your capacity for work? | 1. Very dissatisfied 2. Dissatisfied 3. Neither dissatisfied nor satisfied 4. Satisfied 5. Very satisfied |  |
|  | How satisfied are you with yourself? | 1. Very dissatisfied 2. Dissatisfied 3. Neither dissatisfied nor satisfied 4. Satisfied 5. Very satisfied |  |
|  | How satisfied are you with your personal relationships? | 1. Very dissatisfied 2. Dissatisfied 3. Neither dissatisfied nor satisfied 4. Satisfied 5. Very satisfied |  |
|  | How satisfied are you with your sex life? | 1. Very dissatisfied 2. Dissatisfied 3. Neither dissatisfied nor satisfied 4. Satisfied 5. Very satisfied |  |
|  | How satisfied are you with the support you get from your friends? | 1. Very dissatisfied 2. Dissatisfied 3. Neither dissatisfied nor satisfied 4. Satisfied 5. Very satisfied |  |
|  | How satisfied are you with the conditions of your living place? | 1. Very dissatisfied 2. Dissatisfied 3. Neither dissatisfied nor satisfied 4. Satisfied 5. Very satisfied |  |
|  | How satisfied are you with your access to health services? | 1. Very dissatisfied 2. Dissatisfied 3. Neither dissatisfied nor satisfied 4. Satisfied 5. Very satisfied |  |
|  | How satisfied are you with your mode of transportation? | 1. Very dissatisfied 2. Dissatisfied 3. Neither dissatisfied nor satisfied 4. Satisfied 5. Very satisfied |  |
| The following question refers to **how often** you have felt or experienced certain things n the last in the last two weeks. | | | |
|  | How often do you have negative feelings, such as blue mood, despair, anxiety, depression? | 1. Never 2. Seldom 3. Quite often 4. Very often 5. Always |  |

**Section VII: Mental Health Problems measurement (SRQ_20)**

|  | Do you often have headaches? | 0. No 1. Yes |  |
| --- | --- | --- | --- |
|  | Is your appetite poor? | 0. No 1. Yes |  |
|  | Do you sleep badly? | 0. No 1. Yes |  |
|  | Are you easily frightened? | 0. No 1. Yes |  |
|  | Do your hands shake? | 0. No 1. Yes |  |
|  | Do you feel nervous, tense or worried? | 0. No 1. Yes |  |
|  | Is your digestion poor? | 0. No 1. Yes |  |
|  | Do you have trouble thinking clearly? | 0. No 1. Yes |  |
|  | Do you feel unhappy? | 0. No 1. Yes |  |
|  | Do you cry more than usual? | 0. No 1. Yes |  |
|  | Do you find it difficult to enjoy your daily activities? | 0. No 1. Yes |  |
|  | Do you find it difficult to make decisions? | 0. No 1. Yes |  |
|  | Is your daily work suffering? | 0. No 1. Yes |  |
|  | Are you unable to play a useful part in life? | 0. No 1. Yes |  |
|  | Have you lost interest in things? | 0. No 1. Yes |  |
|  | Do you feel that you are a worthless person? | 0. No 1. Yes |  |
|  | Has the thought of ending your life been on your mind? | 0. No 1. Yes |  |
|  | Do you feel tired all the time? | 0. No 1. Yes |  |
|  | Do you have uncomfortable feelings in your stomach? | 0. No 1. Yes |  |
|  | Are you easily tired? | 0. No 1. Yes |  |

**Amharic Version Questionnaire**

**ክፍል አንድ: የስነ-ህዝብ መረጃ መጠይቆች**

| ተ.ቁ | ጥያቄ | አማራጭ መልሶች |
| --- | --- | --- |
|  | የካርድ ለውጥ |  |
|  | ዞን ------- ወረዳ -------- ቀበሌ--------- | |
|  | እድሜ | ----------- ዓመት |
|  | ጾታ | 1. ሴት 2. ወንድ |
|  | ሃይማኖት | 1. ኦርቶዶክስ 2. ሙስሊም 3. ፕሮቴስታንት 4. ካቶሊክ 5. ሌላ |
|  | መኖሪያ | 1. ከተማ 2. ገጠር |
|  | የጋብቻ ሁኔታ | 1. ያላገባ/ች 2. ያገባ/ች 3. የፈታ/ች 4. የተለያዩ 5. የሞተባት/በት |
|  | የሥራ ሁኔታ | 1. የመንግስት ሰራተኛ 2. በግል ተዳዳሪ 3. ገበሬ 4. ነጋዴ 5. የቤት እመቤት 6. ተማሪ 7. ስራ የሌለው/ላት 8. ሌላ |
|  | የትምህርት ደረጃ | 1. ማንበብና መጻፍ የማይችል 2. ማንበብና መጻፍ የሚችል 3. የመጀመሪያ ደረጃ ትምህርት (1-8) 4. የሁለተኛ ደረጃ ትምህርት (9-12) 5. ዲፕሎማ 6. የመጀመሪያ ዲግሪ ወይም ከዚያ በላይ |
|  | ወርሃዊ ገቢ | --------- ብር |
|  | ከቤተሰብዎ ጋር ያልዎት ግኑኝነት(ከአንድ በላይ መልስ ይቻላል) | 1. የቤተሰብ መሪ 2. እናት 3. አባት 4. ልጅ 5. ዘመድ |
|  | **የህክምናዎ ወጭ የሚሸፈነው በማን ነው**? | 1. በጤና መድን 2. በድህነት ካርድ 3. በግል ወጭ |
|  | **ቤትዎ ከሆስፒታል ምን ያህል ይርቃል**? | -----------ኪ.ሜ |

**ክፍል II: ከህክምና ጋር የተያያዙ ጉዳዮች መጠይቅ**

| **ተ.ቁ** | **መጠይቅ** | **አማራጭ መልሶች** | | |
| --- | --- | --- | --- | --- |
|  | ክትትል ሊያደርጉ የመጡበት በሽታ ምንድን ነው? | 1. ኤ.ች.አይ.ቪ 2. የስኳር በሽታ 3. የደም ግፊት በሽታ 4. ሲ.ኦ.ፒ.ዲ 5. አሰም 6. የጉበት በሽታ 7. የኩላሊት በሽታ 8. የልብ ህመም 9. ካንሰር | | |
|  | በሽታው እንዳለብዎ በሃኪም የተነገረዎ መቼ ነበር ? | (ቀን/ወር/ዓመት)____/ ____/____ | | |
|  | ክትትል ማድረግ ከጀመሩ ምን ያክል ጊዜ ሆነዎት? | ---------ዓመት | | |
|  | ኮሮና ከመምጣቱ በፊት ባለው አንድ ዓመት ውስጥ ያመለጥዎ የክትትል ቀጠሮ ነበር? | 1. አዎ 2. የለም | | |
|  | ለጥያቄ ቁጥር 17 መልስዎ አወ ከሆነ ስንት የክትትል ቀጠሮ ነው ያመለጥዎ (ከቻርት አረጋግጥ)? | ______ | | |
|  | ከኮሮና በፊት ያላቸው የህክምና ውጤት (ቻርት ተመልከት) | 1. በጣም ጥሩ (በመጠነኛ በህክምና ቁጥጥር ውስጥ ያለ) 2. በመጠነኛ የህክምና ቁጥጥር ውስጥ ያለ 3. እየተባባሰ (እያገረሸ) ያለ (ከቁጥጥር ውጭ የሆነ) | | |
|  | ኮሮና ከመጣ ጊዜ ጀምሮ ባለው ጊዜ ያመለጥዎ የክትትል ቀጠሮ ነበር? | 1. አዎ 2. የለም | | |
|  | ለጥያቄ ቁጥር 20 መልስዎ አወ ከሆነ ስንት የክትትል ቀጠሮ ነው ያመለጥዎ (ከቻርት አረጋግጥ)? | ______ | | |
|  | ለጥያቄ ቁጥር 20 መልስዎ አወ ከሆነ ከክትትል ቀጠሮዎ የቀሩበት ምክንያት ምን ነበር? | 1. ለኮሮና ያለኝ ፍራቻ 2. የጤና ተቋማት መዘጋት 3. የጤና ባለሙያዎች ተባባሪ አለመሆን 4. ከኮሮና የአስቸኳይ ጊዜ አዋጅ ጋር ተያይዞ የነበረው የትራንስፖርት ችግር 5. ሌላ ከሆነ ይግለጹ-------- | | |
|  | የኮሮና ወረርሽኝ ከገባ ጊዜ ጀምሮ ባለው ጊዜ የድንገተኛ ህክምና አድርገው ነበር? | 1. አዎ 2. የለም | | |
|  | ሌላ በሃኪም የተረጋገጠ ተጨማሪ በሽታ አለብዎ? | 1. አዎ 2. የለብኝም | | |
|  | ለጥያቄ ቁጥር 24 መልስዎ አወ ከሆነ ከሚከተሉት ውስጥ የትኛው ነው ? (ከአንድ በላይ መልስ ይቻላል) (ከቻርት አረጋግጥ) | 1. ኤ.ች.አይ.ቪ 2. የስኳር በሽታ 3. የደም ግፊት በሽታ 4. ሲ.ኦ.ፒ.ዲ 5. አስም 6. የጉበት በሽታ 7. የኩላሊት በሽታ 8. የልብ ህመም 9. ካንሰር 10. ስትሮክ 11. የሚጥል በሽታ 12. SLE/RA 13. ደም ማነስ 14. ሌላ ከሆነ ይግለጹ | | |
|  | ኮሮና ከገባ ጊዜ ጀምሮ ባለው ጊዜ ከተዘረዘሩት የበሽታ ምልከቶች የትኞቹ ተሰምቶዎት ነበር? | 1. ደረቅ ሳል 2. ከፍተኛ የሰውነት ሙቀት 3. ማሽተት አለመቻል 4. የጡንቻ/የመገጣጠሚያ ህመም 5. ራስ ምታት 6. የትንፋሽ ማጠር | | |
|  | የኮሮና ቫይረስ ወረርሽኝ በምርመራ ተገኝቶቦዎት ነበር? | 1. አዎ 2. የለም | | |
|  | ለጥያቄ ቁጥር 27 መልስዎ አወ ከሆነ የኮሮና ቫይረስ ህክምና ያደረጉት የት ነው? | 1. ቤት 2. ጤና ተቋም | | |
|  | ለጥያቄ ቁጥር 27 መልስዎ የለም ከሆነ ኮሮና ይዞዎት የነበረ ይመስልዎታል? | 1. በፍጹም አይመስለኝም 2. አይመስለኝም 3. መወሰን አልችልም 4. ይመስለኛል 5. በጣም ይመስለኛል | | |
|  | የሚወስዱት የመዳኅኒት አይነቶች ምን ምን ናቸው?(ከአንድ በላይ መልስ ይቻላል) | 1. የሚዋጥ 2. በመርፌ የሚሰጥ 3. ሁለቱንም አይነት 4. በአፍንጫ የሚነፋ 5. ሁሉንም | | |
|  | በቀን ውስጥ ስንት ጊዜ መድሃኒት ይወስዳሉ? | ---------- | | |
|  | ስንት ዓይነት መድሃኒት/ቶች ይወስዳሉ? | --------- | | |
|  | የክትትል ቀጠሮዎ በየስንት ጊዜ ነው? | 1. በየሳምንት ወይም ከዛ ባነሰ 2. በየሁለት/ሶስት ሳምንት 3. በየወሩ 4. በየሁለት ወር 5. በየሦስት-አምስት ወር 6. በየስድስት ወር ወይም ከዚያ በላይ | | |
|  | ካለብዎ ህመም ጋር የተያያዘ የጤና መቃዎስ አለብዎ? | 1. አዎ 2. የለብኝም | | |
|  | ለQ34 መልስዎ አዎ ከሆነ ምን እንደሆነ ይግለጹ? | ______________ | | |
|  | ኮሮና ከገባ በኋላ ከመጋቢት-ግንቦት ባሉት 3 ወራት ውስጥ በነበራቸው መስከረም-ኅዳር | 1 | 2 | 3 |
|  | ቀጠሮ 1 (ቀን/ወር/ዓመት) -------/--------/--------- |  |  |  |
|  | ቀጠሮ 2 (ቀን/ወር/ዓመት) -------/--------/--------- |  |  |  |
|  | ቀጠሮ 3 (ቀን/ወር/ዓመት) -------/--------/--------- |  |  |  |
|  | ቀጠሮ 4 (ቀን/ወር/ዓመት) -------/--------/--------- |  |  |  |

**ማስታወሻ፡ 1** =በጣም ጥሩ (በህክምና ቁጥጥር ውስጥ ያለ) 2 = በመጠነኛ የህክምና ቁጥጥር ውስጥ ያለ 3= እየተባባሰ (እያገረሸ) ያለ (ከቁጥጥር ውጭ የሆነ)

**ክፍል ሶስት፡ የመዳሕኒት አወሳሰድ (Morisky Medication Adherence Scale)**

| ተ.ቁ | ጥያቄ | መልስ |  |
| --- | --- | --- | --- |
|  | መድሃኒት መውሰድዎን የሚረሱበት ጊዜ አለ? | 1. አዎ 2. የለም |  |
|  | ባለፉት ሁለት ሳምንታት መድሃኒት ያልወሰዱበት ቀን አለ? | 1. አዎ 2. የለም |  |
|  | ህመሙ ሲብስብዎ ለሐኪምዎ ሳይነገሩ መድሃኒትዎን አቁመው ያውቃሉ? | 1. አዎ 2. የለም |  |
|  | አንዳንድ ጊዜ መንገድ ሲወጡ ወይም ከቤት ለመውጣት ሲያስቡ መድሃኒትዎን መያዝዎን ይረሳሉ? | 1. አዎ 2. የለም |  |
|  | ትላንትና የታዘዘልዎን መድሃኒት በሙሉ  ወስደዋል? | 1. አዎ 2. የለም |  |
|  | የበሸታዎ ምልክቶች በህክምና ቁጥጥር መጥፋት ሲጀምሩ, አንዳንዴ መድሃኒት መውሰድዎን ያቋርጣሉ? | 1. አዎ 2. የለም |  |
|  | በየቀኑ መድሃኒት መውሰዶ ይረብሾታል/ ያስቸግሮታል? | 1. አዎ 2. የለም |  |
|  | ሁሉንም መድሃኒትዎን አስታውሰው ለመውሰድ ምን ያህል ጊዜ ይቸገራሉ? | 1. በጭራሽ 2. ከስንት አንድ ጊዜ 3. አንዳንድ ጊዜ 4. ብዙ ጊዜ 5. ሁልጊዜ |  |

**ክፍል አራት: ማህበራዊ ድጋፍ**

| ተ.ቁ | ጥያቄ | አማራጭ ምላሽ | እለፍ |
| --- | --- | --- | --- |
|  | ትልቅ ችግር ሲያጋጥምዎ ሊረድዎ የሚችሉ በዙሪያዎ ምን ያህል ሰዎች አሉ? | 1. ማንም የለኝም 2. 1-2 ሰዎች 3. 3-5 ሰዎች 4. 5ና ከዚያ በላይ |  |
|  | በዙሪያዎ ያሉ ሰዎች ስለርስዎ ምን ያህል እሚያስቡልዎት ይመስልዎታል? | 1. ፈጽሞ አያስቡልኝም 2. አያስቡልኝም 3. እርግጠኛ አይደለሁም 4. ያስቡልኛል 5. በጣም ያስቡልኛል |  |
|  | እርዳታ በሚያስፈልግዎ ጊዜ ከጎረቤትዎ የማገኘት እድልዎ ምን ያህል ነው? | 1. በጣም ከባድ ነው 2. ከባድ ነው 3. መጠነኛ ነው 4. ቀላል ነው 5. በጣም ቀላል ነው |  |

**ክፍል አምስት፡ ከባህርይ ጋር ተያያዘዥ የሆኑ ነገሮች መጠይቅ**

- 1. **የአልኮል አጠቃቀም**

| **ተ.ቁ** | **ጥያቄ** | የመልስ አማራጮች |
| --- | --- | --- |
|  | ምን ያህል ጊዜ አልኮል የያዘ መጠጥ ጠጥተዉ ያዉቃሉ? | 0. በጭራሽ [ወደ ሚቀጥለው ክፍል ይሂዱ]  1. በየወሩ ወይም ከዚያ ባነሰ  2. በወር ከ 2 እስከ 4 ጊዜ  3. በሳምንት ከ 2 እስከ 3 ጊዜ  4. በሳምንት 4 ወይም ከዚያ በላይ ጊዜ |
|  | የሚጠጡት የመጠጥ አይነት ምንድን ነው? | 1. ቢራ 2. ጠላ 3. አረቄ 4. ወይን 5. ጠጅ 6. ሌላ ከሆነ ይግለጹ------------------- |
|  | በአንድ ቀን ስንት ብርሌ ጠጅ ይጠጣሉ? | _____ በትንሹ ጣሳ ጠላ/ኮረፌ  -------ጠርሙስ ቢራ  -------መለኪያ አረቄ/ውስኪ  -------ብርጭቆ ወይን  --------ብርሌ ጠጅ |
|  | ለምን ያክል ጊዜ በአንድ ቀን 6 (ለሴት)/8(ለወንድ) እና ከዚያ በላይ መጠጦች ይጠጣሉ? | 0. በጭራሽ  1. ከወር በታች  2. በየወሩ  3. በየሳምንቱ  4. በየቀኑ ወይም በየቀኑ ማለት ይቻላል |
|  | ባለፈዉ አመት መጠጥ ጀምረዉ ለማቆም ምን ያህል ጊዜ ተቸግረዋል? | 0. በጭራሽ  1. ከወር በታች  2. በየወሩ  3. በየሳምንቱ  4. በየቀኑ ወይም በየቀኑ ማለት ይቻላል |
|  | ባለፈዉ ዓመት ዉስጥ መስራት ያለበዎትን ስራ በመጠጥ ምክንያት ምን ያክል ሳያሳኩ ቀርተዋል? | 0. በጭራሽ  1. ከወር በታች  2. በየወሩ  3. በየሳምንቱ  4. በየቀኑ ወይም በየቀኑ ማለት ይቻላል |
|  | ባለፈው ዓመት ዉስጥ ብዙ መጠጥ ጠጥተዉ ካደሩ በኋላ ቀንዎን ለመጀመር ጠዋት መጠጥ ምን ያህል ጊዜ ፈልገዋል? | 0. በጭራሽ  1. ከወር በታች  2. በየወሩ  3. በየሳምንቱ  4. በየቀኑ ወይም በየቀኑ ማለት ይቻላል |
|  | ባለፈው ዓመት ውስጥ ምን ያህል ጊዜ ከጠጡ በኋላ የጥፋተኝነት ስሜት ተሰምቶዎታል? | 0. በጭራሽ  1. ከወር በታች  2. በየወሩ  3. በየሳምንቱ  4. በየቀኑ ወይም በየቀኑ ማለት ይቻላል |
|  | ባለፈው ዓመት ምን ያህል ጊዜ በመጠጣትዎ ምክንያት ማታ ምን እንደ ተፈጠረ ለማስታወስ አልቻሉም? | 0. በጭራሽ  1. ከወር በታች  2. በየወሩ  3. በየሳምንቱ  4. በየቀኑ ወይም በየቀኑ ማለት ይቻላል |
|  | በመጠጣትዎ ምክንያት በእርስዎ ወይም ሌላ ሰው ላይ ጉዳት ደርሶ ያውቃል? | 0. የለም  1. አዎ፣ ግን ባለፈዉ አንድ አመት አይደለም  2. አዎ፣ ባለፈዉ አንድ አመት |
|  | ዘመድዎ ፣ ጓደኛዎ ፣ ሐኪምዎ ወይም ሌላ የጤና ሠራተኛ ስለ መጠጣትዎ ተጨንቆ እንዲያቆሙ ነግሮዎት ያዉቃል? | 0.የለም  1. አዎ፣ ግን ባለፈዉ አንድ አመት አይደለም  2. አዎ፣ ባለፈዉ አንድ አመት |

- 1. **ሲጋራ አጠቃቀም**

| **ተ.ቁ** | **ጥያቄ** | **ምላሽ** |
| --- | --- | --- |
|  | ሲጋራ አጭሰዉ ያውቃሉ? | 1.አዎ  2. የለም |
|  | ለ ጥያቄ ቁ.63 አዎ ከሆነ ባለፈው አንድ ወር ውስጥ አጭሰው ነበር? | 1.አዎ  2. የለም |
|  | ለጥያቄ ቁ.64 አዎ ከሆነ ባለፈው ወር ውስጥ ስንት ጊዜ አጨሰዋል? | ----- |
|  | በአማካይ በቀን ስንት ሲጋራ ያጨሳሉ? | --------- |
|  | የተለየ ሲጋራ አይነት ያጨሳሉ? | 1.አዎ  2. የለም |
|  | ለጥያቄ ቁ.67 አዎ ከሆነ የሚያጨሱት የሲጋራ አይነት የቱ ነው? | 1. ፓይፕ 2. እሚታኝክ 3. በአፍንጫ እሚወሰድ 4. ሽሻ 5. ጋያ ሌላ ከሆነ ይግለጹ |
|  | በቤትዎ ውስጥ ሲጋራ የሚያጨስ ሰው አለ? | 1.አወ  2. የለም |
| **የጫት አጠቃቀም** | | |
|  | ጫት ተጠቅመው ያውቃሉ | 1.አወ  2. የለም |
|  | ለጥያቄ ቁ.70 መልስዎ አዎ ከሆነ ባለፈው ወር ውስጥ ተጠቅመዋል? | 1.አወ  2. የለም |
|  | ለጥያቄ ቁ.71 መልስዎ አዎ ከሆነ ባለፈው ወር ውስጥ ለስንት ቀናት ተጠቅመዋል? | --------ቀን(ናት) |

- 1. **የአካል ብቃት እንቅስቃሴ መጠይቅ**

| *ተ.ቁ* | *ጥያቄ* | *አማራጭ መልሶች* | Code |
| --- | --- | --- | --- |
|  | ሥራዎ ቢያንስ ለ10 ደቂቃዎች ያለማቋረጥ የትንፋሽ ወይም የልብ ምትን በከፍተኛ ሁኔታ ሊጨምሩ የሚችሉ ማለትም ከባድ ሸክሞችን መሸከም ወይም ማንሳት ፣ መቆፈርና የግንባታ ሥራ ወዘተ የመሳሰሉትን እንቅስቃሴን ያካትታል? | 1. አወ 2. የለም   *መልስዎ የለም ከሆነ ወደ* Q4 *ይሂዱ* | Q1 |
|  | በሳምንት ውስጥ በስራዎ ምክንያት ከባድ የአካል ብቃት እንቅስቃሴዎችን ለስንት ቀናት ያካሂዳሉ? | _________ቀን | Q2 |
|  | ቀን ውስጥ በሥራዎ ምክንያት ለምን ያህል ጊዜ ከባድ የአካል ብቃት እንቅስቃሴዎችን ይሰራሉ? | ________ደቂቃ | Q 3 |
|  | ሥራዎ መጠነኛ እንቅስቃሴዎችን ማለትም እንደ ፈጣን የእግር ጉዞ ወይም ቀላል ሸክሞችን ቢያንስ ለ 10 ደቂቃዎች ያለማቋረጥ መስራትን ያካትታል? | 1. *አወ* 2. *አላደርግም*   *መልስዎ አላደርግም ከሆነ ወደ* Q7 *ይሂዱ* | Q 4 |
|  | በሳምንት ውስጥ በስራዎ ምክንያት መጠነኛ የአካል ብቃት እንቅስቃሴዎችን ለስንት ቀናት ያካሂዳሉ? | _________ቀን | Q5 |
|  | በቀን ውስጥ በሥራዎ ምክንያት ለምን ያህል ጊዜ መጠነኛ የአካል ብቃት እንቅስቃሴዎችን ይሰራሉ? | ________ደቂቃ | Q6 |
|  | ከቦታ ወደ ቦታ ለመሄድ በእግርዎ ወይም በብስክሌት ቢያንስ ለ 10 ደቂቃዎች ጉዞ ያደርጋሉ? | 1. አወ 2. አላደርግም   *መልስዎ አላደርግም ከሆነ ወደ* Q10 *ይሂዱ* | at Q7 |
|  | በሳምንት ውስጥ በተከታታይ ቢያንስ ለ 10 ደቂቃዎች ስንት ቀናት በእግር ወይም በብስክሌት ይጓዛሉ? | …….ቀን | Q8 |
|  | በቀን ውስጥ በእግር ወይም በብስክሌት በመጓዝ ምን ያህል ጊዜ ያሳልፋሉ?  (በደቂቃ) | ________ | Q 9 |
|  | በከፍተኛ ሁኔታ የትንፋሽ ወይም የልብ ምትን መጨመር የሚያስከትሉ ከባድ የአካል ብቃት እንቅስቃሴዎችን (ለምሳሌ፡- ክብደት ማንሳት፣ ረጅም ሩጫ፣ ፑሽ አፕ፣ ፑል አፕ የመሳሰሉትን) ያለማቋረጥ ቢያንስ ለ 10 ደቂቃዎች ያደርጋሉ? | 1. *አወ* 2. *አላደርግም*   *መልስዎ አላደርግም ከሆነ ወደ* Q13 *ይሂዱ* | Q10 |
|  | በሳምንት ውስጥ ከባድ የአካል ብቃት እንቅስቃሴዎችን ለስንት ቀናት ያደርጋሉ? | _________ቀን | Q11 |
|  | በቀን ውስጥ ከባድ የአካል ብቃት እንቅስቃሴዎችን ለምን ያህል ጊዜ ይሰራሉ?(በደቂቃ) | ________ደቂቃ | Q12 |
|  | መጠነኛ ማለትም እንደ ገመድ መዝለል ፣ የሶምሶማ ሩጫ ፣ ውሃ ዋና ያሉ የአካል ብቃት እንቀስቃሴዎችን ያለማቋረጥ ቢያንስ ለ10 ደቂቃዎች ይሰራሉ? | 1. *አወ* 2. *አልሰራም*   *መልስዎ አላደርግም ከሆነ ወደ* Q16 *ይሂዱ* | Q13 |
|  | በሳምንት ውስጥ ከላይ የተጠቀሱትን መጠነኛ የአካል ብቃት እንቅስቃሴዎችን ለስንት ቀናት ያካሂዳሉ? | _________ቀን | Q 14 |
|  | በቀን መጠነኛ የአካል ብቃት እንቅስቃሴዎችን ለምን ያህል ጊዜ ይሰራሉ?(በደቂቃ) | __________ደቂቃ | Q 15 |
| Sedentary behaviour | | |  |
|  | በቀን ውስጥ በመቀመጥ ወይም ጋደም ብለው ምን ያህል ጊዜ ያሳልፋሉ? (በሰዓት) | _________ደቂቃ | Q16 |

**ከፍፍል አምስት፡ Health Related Quality Life**

|  | የህይወትዎን/አኗኗርዎን አንዴት ይመዝኑታል? | 1. በጣም ዝቅተኛ 2. ዝቅተኛ 3. መካከለኛ 4. ጥሩ 5. በጣም ጥሩ |  |
| --- | --- | --- | --- |
|  | በጤንነትዎ ምን ያህል ረክተዋል? | 1. በጭራሽ 2. በትንሽ 3. መካከለኛ 4. በጣም 5. እጅግ በጣም |  |
| የሚከተሉት ጥያቄዎች ባለፉት ሁለት ሳምንታት ውስጥ ስላጋጠሟችሁ አንዳንድ ነገሮች የሚዳስሱ መጠይቆች ናቸው፡፡ | | | |
|  | በምን ያህል መጠን የአካላዊ ህመም የዕለት-ከዕልት ስራወትን እንዳይሰሩ ከልክሎታል? | 1. በጭራሽ 2. በትንሽ 3. መጠነኛ 4. በጣም 5. እጅግ በጣም |  |
|  | የዕለት ተዕለት እንቅስቃሴዎ እንዳይጓደል ምን ያህል የህክምና ዕርዳታ ያስፈልግዎታል? | 1. በጭራሽ 2. በትንሽ 3. መጠነኛ 4. እጅግ በጣም 5. እጅግ በጣም |  |
|  | በህይወትዎ ምን ያህል ይደሰታሉ? | 1. በጭራሽ 2. በትንሽ 3. በመጠኑ 4. በጣም 5. እጅግ በጣም |  |
|  | ህይወትዎ/አኗኗርዎን ምን ያህል ትርጉም አለው ብለው  ያስባሉ? | 1. በጭራሽ 2. በትንሽ 3. በመጠኑ 4. በጣም 5. እጅግ በጣም |  |
|  | ሀሳበዎን ለማሰባሰብ ምን ያህል አቅም አለዎት? | 1. በጭራሽ 2. በትንሽ 3. መጠነኛ 4. በጣም 5. እጅግ በጣም |  |
|  | በዕለታዊ ህይወትዎ ምን ያህል ደህንነት ይሰማዎታል? | 1. በጭራሽ 2. በትንሽ 3. መጠነኛ 4. በጣም 5. እጅግ በጣም |  |
|  | የሚኖሩበት አካባቢ ምን ያህል ጤናማ/ምቹ ነው? | 1. በጭራሽ 2. በትንሽ 3. መጠነኛ 4. በጣም 5. እጅግ በጣም |  |
| የሚከተሉት ጥያቄዎች ባለፉት ሁለት ሳምንታት ውስጥ ምን ምን ነገሮችን ሙሉ በሙሉ እንደፈጸሙ ወይም ለማድረግ ሙከራ አደረጉ የሚሉu ጉዳዮችን የመሚጠይቁ ናቸው፡፡ | | | |
|  | ለዕለት ተዕለት እንቅስቃሴዎ በቂ ጉልበት/አቅም አለዎት? | 1. በጭራሽ 2. ትንሽ 3. በመጠኑ 4. በአብዛኛው 5. ሙሉ በሙሉ |  |
|  | አሁን ያለዎትን የሰውነትዎን ቁመና ምና ያህል ይቀበሉታል? | 1. በጭራሽ 2. ትንሽ 3. በመጠኑ 4. በአብዛኛው 5. ሙሉ በሙሉ |  |
|  | ፍላጎቶችዎን ለማሟላት የሚያስችል በቂ ገንዘብ አለዎት? | 1. በጭራሽ 2. ትንሽ 3. በመጠኑ 4. በአብዛኛው 5. ሙሉ በሙሉ |  |
|  | ለዕለት ተዕለት ህይዎትዎ አስፈላጊዉን መረጃ ያገኛሉ? | 1. በጭራሽ 2. ትንሽ 3. በመጠኑ 4. በአብዛኛው 5. ሙሉ በሙሉ |  |
|  | ለመዝናናት (ቅንጦት) ምን ያህል እድል አለዎት? | 1. በጭራሽ 2. ትንሽ 3. በመጠኑ 4. በአብዛኛው 5. ሙሉ በሙሉ |  |
|  | ከቦታ ወደ ቦታ በቀላሉ ለምዘዋወር የሚያስችል ጤንነት አለዎት? | 1. በጭራሽ 2. ትንሽ 3. በመጠኑ 4. በአብዛኛው 5. ሙሉ በሙሉ |  |
| የሚከተሉት ጥያቄዎች ባለፉት ሁለት ሳምንታት ውስጥ ስለህይወትዎ የተለያዩ ገጽታዎች ምን ያህል እንደተሰማዎት ወይም እንደረካዎ እንዲናገሩ የሚጠይቁ ናቸው ፡ | | | |
|  | በእንቅልፍዎ ምን ያህል ረክተዋል? | 1. በጣም አልረካሁም 2. አልረካሁም 3. መካከለኛ 4. ረክቻለሁ 5. በጣም ረክቻለሁ |  |
|  | የዕለት ተዕለት ህይወተወን ለመምራት በአቅመዎ ምን ያህል ረክተዋል? | 1. በጣም አልረካሁም 2. አልረካሁም 3. መካከለኛ 4. ረክቻለሁ 5. በጣም ረክቻለሁ |  |
|  | ለሥራ ባለወት አቅም ምን ያህል ረክተዋል? | 1. በጣም አልረካሁም 2. አልረካሁም 3. መካከለኛ 4. ረክቻለሁ 5. በጣም ረክቻለሁ |  |
|  | በራስዎ ምን ያህል ረክተዋል? | 1. በጣም አልረካሁም 2. አልረካሁም 3. መካከለኛ 4. ረክቻለሁ 5. በጣም ረክቻለሁ |  |
|  | ከሰዎች ጋር ባለዎት ግንኙነትዎ ምን ያህል ረክተዋል? | 1. በጣም አልረካሁም 2. አልረካሁም 3. መካከለኛ 4. ረክቻለሁ 5. በጣም ረክቻለሁ |  |
|  | በወሲብ ሕይወትዎ ምን ያህል ረክተዋል? | 1. በጣም አልረካሁም 2. አልረካሁም 3. መካከለኛ 4. ረክቻለሁ 5. በጣም ረክቻለሁ |  |
|  | ከጓደኞችዎ በሚያገኙት ድጋፍ ምን ያህል ረክተዋል? | 1. በጣም አልረካሁም 2. አልረካሁም 3. መካከለኛ 4. ረክቻለሁ 5. በጣም ረክቻለሁ |  |
|  | በመኖሪያ ቦታዎ ምን ያህል ረክተዋል? | 1. በጣም አልረካሁም 2. አልረካሁም 3. መካከለኛ 4. ረክቻለሁ 5. በጣም ረክቻለሁ |  |
|  | በጤና አገልግሎት አቅርቦት ምን ያህል ለክተዋል? | 1. በጣም አልረካሁም 2. አልረካሁም 3. መካከለኛ 4. ረክቻለሁ 5. በጣም ረክቻለሁ |  |
|  | በመጓጓዣ ዘዴዎ ምን ያህል ረክተዋል? | 1. በጣም አልረካሁም 2. አልረካሁም 3. መካከለኛ 4. ረክቻለሁ 5. በጣም ረክቻለሁ |  |
| የሚከተለው ጥያቄ የሚያመለክተው ባለፉት ሁለት ሳምንታት ውስጥ አንዳንድ ነገሮች ምን ያህል ጊዜ እንደተሰማዎት ወይም እንደገጠሙዎት ነው ፡፡ | | | |
|  | ምን ያህል በተደጋጋሚ አሉታዊ ስሜቶች ማለትም እንደ መከፋት፤ ተስፋ መቁረጥ ጭንቀት ወይም ድብርተ ደረሶብዎት ያውቃል? | 1. በጭራሽ 2. አልፎ አልፎ 3. ብዙ ጊዜ 4. በተደጋጋሚ 5. ሁል ጊዜ |  |

**ክፍል ስድስት፡** የአእምሮ ህመም መለኪያ መጠይቅ

| **ተ.ቁ** | **ጥያቄ** | **መልስ** | |
| --- | --- | --- | --- |
|  | ተደጋጋሚ እራስ ምታት አለዎት? | 1. አዎ | 1. የለም |
|  | የምግብ ፍላጎትዎ ቀንሷል? | 1. አዎ | 1. የለም |
|  | የእንቅልፍ ችግር አለብዎት? | 1. አዎ | 1. የለም |
|  | ፍርሀት ፍርሀት ይልዎታል? | 1. አዎ | 1. የለም |
|  | እጅዎ ይንቀጠቀጣል? | 1. አዎ | 1. የለም |
|  | መረበሽ፣ መጨነቅና ውጥረት ይሰማዎታል? | 1. አዎ | 1. የለም |
|  | ምግብ አልፈጭ ብሎዎታል? | 1. አዎ | 1. የለም |
|  | በትኩረት ለማሰብ ይቸገራሉ? | 1. አዎ | 1. የለም |
|  | ያለመደሰት ስሜት አለዎት? | 1. አዎ | 1. የለም |
|  | በተደጋጋሚ ያለቅሳሉ? | 1. አዎ | 1. የለም |
|  | የእለት ተእለት ሥራዎን ለማከናወን ተቸግረዋል? | 1. አዎ | 1. የለም |
|  | ለመወሰን ተቸግረዋል? | 1. አዎ | 1. የለም |
|  | በቀን ተቀን ስራዎ ላይ ችግር አጋጥሞዎታል? | 1. አዎ | 1. የለም |
|  | በኑሮዎ የሚጠበቅብዎትን ሓላፊነት ለመወጣት ተቸግረዋል? | 1. አዎ | 1. የለም |
|  | በሁሉም ነገር ፍላጎት አጥተዋል? | 1. አዎ | 1. የለም |
|  | ዋጋ የለኝም የሚል ስሜት አለዎት? | 1. አዎ | 1. የለም |
|  | ራስዎን የማጥፋት ሃሳብ መጥቶብዎ ያውቃል? | 1. አዎ | 1. የለም |
|  | ሁልጊዜ ድካም ስሜት ሰማዎታል? | 1. አዎ | 1. የለም |
|  | በቀላሉ ይደክምዎታል? | 1. አዎ | 1. የለም |
|  | ሆድዎ ይታወካል ወይም ምቾት አይሰማዎትም?  ባለፉት ሶስት ወራት ውስጥ የስኳር ማነስ ምልክቶች ማለትም ከፍተኛ የልብ ምት መጨመር፣ ላብ ላብ ማለት፣ የንቃተ ህሊና መቃወስ ወይም የደምዎ የስኳር መጠን ከ 80ሚግ/ዲኤል መሆን)አጋጥሞዎት ነበር ? | 1. አዎ | 1. የለም |
