# Supplementary material for: Effect of COVID-19 pandemic on missed medical appointment among adults with chronic disease conditions in Northwest Ethiopia
Source: PLoS One. 2022 Oct 4;17(10):e0274190. doi: 10.1371/journal.pone.0274190 (PMC9531804; doi:10.1371/journal.pone.0274190)
Supplement: Attachment — Submitted filename: Response to Reviewers.docx [file pone.0274190.s003.docx]

May 2022

**Rebuttal letter**

**Manuscript ID:**

PONE-D-21-28858

**Title:** Effect of COVID-19 pandemic on missed medical appointments among adults with chronic diseases conditions in Northwest, Ethiopia

*Tadesse Awoke Ayele, Tesfa Sewunet Alamneh, Habtewold Shibru, MaledeMequanent Sisay, Tesfahun MeleseYilma, MelkituFentie Melak, Telake Azale Bisetegn, Tariku Belachew, Mahteme Haile, Taye Zeru,Mezgebu SelamsewAsres, and Kegnie Shitu*

**Dear Editor and reviewer,**

We would like to thank for your consideration and suggestion for the betterment of our manuscript and make it more informative. We tried to amend the format of the manuscript according to the journal guidelines and address the questions raised by reviewer on the manuscript. The authors revised the overall manuscript regarding to language usage and grammar errors. In addition, we also consult language experts in our university and amendments were done based on their comments. Our point-by-point responses for each comment and questions are described in detail on the following pages. Further, the details of changes were shown by track changes in the supplementary document attached.

Editor’s comment

1. Please ensure that your manuscript meets PLOS ONE's style requirements, including those for file naming. The PLOS ONE style templates can be found at<https://journals.plos.org/plosone/s/file?id=wjVg/PLOSOne_formatting_sample_main_body.pdf> and<https://journals.plos.org/plosone/s/file?id=ba62/PLOSOne_formatting_sample_title_authors_affiliations.pdf>

**Authors’ response:** Thank you dear editor for your concern. We tried to adjust the format according to the journal requirements

2. You indicated that you had ethical approval for your study. In your Methods section, please ensure you have also stated whether you obtained consent from parents or guardians of the minors included in the study or whether the research ethics committee or IRB specifically waived the need for their consent..

**Authors’ response:** Thank you dear editor for your concern. The study participants were individuals who are aged 18 years and above. Therefore, they can give informed consents by themselves and we don’t include other minority groups.

3. Please include additional information regarding the survey or questionnaire used in the study and ensure that you have provided sufficient details that others could replicate the analyses. For instance, if you developed a questionnaire as part of this study and it is not under a copyright more restrictive than CC-BY, please include a copy, in both the original language and English, as Supporting Information.

**Authors’ response:** Thank you dear editor for your concern. We include it as supporting information.

4. We note that you have indicated that data from this study are available upon request. PLOS only allows data to be available upon request if there are legal or ethical restrictions on sharing data publicly. For more information on unacceptable data access restrictions, please see <http://journals.plos.org/plosone/s/data-availability#loc-unacceptable-data-access-restrictions>.

**Authors’ response:** Thank you dear editor for your concern. We have presented the appropriate data availability statement on the online submission and upload de-identified dataset.

5. Your ethics statement should only appear in the Methods section of your manuscript. If your ethics statement is written in any section besides the Methods, please delete it from any other section.

**Authors’ response:** Thank you dear editor for your concern. We have put it at the end of methods section as per your recommendation.

**To reviewer 1**

**Reviewer comments**

**Abstract:**

1. Results: There seems to be a missing word where it reads ‘most (55.4%) were female.’ Suggest need to add in ‘participants’ or similar.

**Authors’ response:** Thank you dear reviewer for your concern. Yes, we can’t say most instead of more than half of the study participants. To make the abstract short and precise we have excluded this statement from the abstract because interested readers can catch it from the result section

2. Conclusion: This is repetitive as it duplicates the reasons of missed appointments which are also found in results. The conclusions would be better to include more about the implications of the findings or the next steps for future research. This relates to a later comment about the conclusion in the main body of the paper.

**Authors’ response:** Thank you dear reviewer for your concern. We include the implications of the findings in the revised documents.

**Introduction:**

3. The first paragraph of the introduction would benefit from more references. If the references that are provided also cover earlier points, it might be worth adding the reference number in again. Some points seem to be unreferenced so this would make it clearer. One example of this is ‘Access to health facilities was restricted due to fear of the spread of the virus from patient and health professional perspectives. Patients were scared to go to a health facility to receive their care and treatments.’ This occurs throughout the introduction.

**Authors’ response:** Thank you dear reviewer for your concern. We add revised this paragraph and include additional reference.

4. In the second paragraph of the introduction you state that ‘Although the virus infects every individual’ this would read better as ‘potentially infects every individual’ as much is still unknown and not everyone is infected.

**Authors’ response:** Thank you dear reviewer for your concern. We have edited it as per your comments.

5. When you say ‘An estimated 41% of adults had delayed or avoided medical care including urgent or emergency care (12%) and routine care (32%) because of concerns about COVID-19 in the US’ it would be helpful to define the timeframe that this covers.

**Authors’ response:** Thank you dear reviewer for your concern. We have included the time frame.

6. It would be useful to include a statement on why looking at missed appointments is needed/ relevant/ interesting. What is the impact of missed appointments on health services and patients? (cost and time for health care professionals and unmet need/ delayed treatment for patients).

**Authors’ response:** Thank you dear reviewer for your concern. We have included an statement entertain what you raised in the last paragraph of the introduction in the revised document.
**Methods:**

7. Study design and period: ‘Two years retrospective data’ could you just briefly state what type of data this was- e.g. was it electronic patient records etc?
   **Authors’ response:** Thank you dear reviewer for your concern. We used two year data that were extracted from chart and some data were collected by the interview at the time of data collection
8. Study setting: Of the 9 hospitals that participated in the study, what were their demographics? (e.g. were they rural/ urban etc).

**Authors’ response:** Thank you dear reviewer for your concern. They are located at urban areas and there deference is level of service they provide but they serve peoples from both urban and rural areas.

**Data collection methods and measurement:**

9. This sentence would be worth rewording to make it clearer: ‘During data collection, it has been about a year since the emergence of the COVID-19 pandemic.’ Something like ‘Data collection was conducted approximately a year into the pandemic.’

**Authors’ response:** Thank you dear reviewer for your concern. We rewrite it in the main document.

10. On page 7 you mention patient interviews. This is the first mention, and it is not clear what this relates to. Is this a reference to the surveys that were used? It implies that there is a qualitative component to the study, so suggest rewording it.
    **Authors’ response:** Thank you dear reviewer for your concern. We used both primary and secondary data source to answer our research questions. We extract a two year data from chart but there are some variables that were not recorded at the chart. Then we conduct patient interview to collect data for variables that are not found in patient chart.
    **Results:**
11. Socio-demographic characteristics: In line with earlier comment, when you say ‘A total of 1,833 individuals with common chronic diseases were interviewed’ do you mean ‘A total of 1,833 individuals with common chronic diseases completed the survey.’ Possibly reword to make it clearer they completed the survey.
    **Authors’ response:** Thank you dear reviewer for your concern. We have edited it in the revised version.
12. ‘More than one-fourth (28.0%) of the individuals with a common chronic condition was not able to read and write’ should say ‘were’ not ‘was.’
    **Authors’ response:** Thank you dear reviewer for your concern. We have corrected it in the revised document.
13. There are a few examples in the results sections where the language would benefit from being revised to make it clearer. E.g. ‘About one in four patients (23.63%) have been following for more than 10 years,’ (have been receiving follow-up?) and ‘However, only 33 (1.8%) diagnoses for COVID-19 and all of them were positive,’ (Diagnosed implies that they were positive) ‘Nearly one-third (33.35%) of the patients had identified co-morbid diseases in which hypertension was the commonest type of co-morbidity observed in most of the patients.’ (Duplication- commonest and most- suggest just use one or the other).
    **Authors’ response:** Thank you dear reviewer for your concern. We have checked the overall manuscript regarding language usage and grammar errors as and we try to amend it. In addition, we also consult language experts in our university and amendments were done based on their comments.
14. Missed appointments: Again a few suggestions on improving the language. E.g. ‘About 225 (12.47% (95% CI: 11.13%, 14.20%)) of patients with common chronic diseases have missed their appointment during their follow-up before COVID 19 pandemic’ (remove ‘have’).
    **Authors’ response:** Thank you dear reviewer for your concern. We have removed it
    **Discussion:**
15. You start by discussing generally about Covid and low-income countries, but then move on to talk specifically about the country being studied. ‘There have been different strategies and non-pharmaceutical interventions implemented, however, the implementation level has been very limited due to the cultural and traditional practices in the country’. It would be good to state here when you move on to talk about one specific country to make it clear.

**Authors’ response:** Thank you dear reviewer for your concern. We have modified it as per your comment.

16. Again a few missed words in this section ‘the rate of COVID-19 transmission is higher United States, Singapore, Canada and Italy.’ Missing ‘in’.
    **Authors’ response:** Thank you dear reviewer for your concern. We include it.
17. Strengths and limitations: There is limited discussion of the strengths and limitations for this study. You mention a few strengths but no discussion of why they are strengths or how they strengthen the study.
    **Authors’ response:** Thank you dear reviewer for your concern. We revise the strength and limitations of the study.
18. I also suggest you could include a section on implications and discuss how this research can help understanding of this topic, implementation of policy or impact on practice. I also think that it would be useful to include any future research needed or [planned on the topic. This also needs to be included in the abstract.
    **Authors’ response:** Thank you dear reviewer for your concern. We include the implication of the study in the both abstract and conclusion section.

Reviewer 2:

Reviewer comments

1. The authors conducted a retrospective cohort study to evaluate the impact of the COVID-19 pandemic on missed appointments among those with chronic conditions in Northwest of Ethiopia. It was important to know the scale of the impact and the risk factors that were associated with increased risk of no-show. Here are my concerns: I do not know why the author called their study “chart review and cross-sectional survey”. Their data source was electronic chart. I also did not see any conventional survey was conducted. Suggest remove “chart review and cross-sectional survey” and replace it with “a retrospective cohort study”.

**Authors’ response:** Thank you dear reviewer for your concern. The data source for this study was primary data which was collected cross-sectionally by interview at the time of their hospital visit and secondary data was extracted from patient chart. From the patient chart, we extract 2 years data one year before the COVID-19 pandemic and a year after the pandemic but we only assess two years data retrospectively but we didn’t have control groups. Therefore, retrospective cohort study might not be appropriate for this scenario because we didn’t have control group.

2. Appointment data were extracted during the COVID-19 pandemic and one year before the pandemic for the same patients. Thus, the outcome, missed appointments from these two periods were not independent. However, the authors used an independent t-test to test the difference in missed appointments before and during the pandemic. In addition, t-test may not be the right test for the number of missed appointments. A binomial test is preferred for count data, or just run the negative binomial regression model without including covariates.

**Authors’ response:** Thank you dear reviewer for your concern. The t-test was conduced to assess the proportion of missed appointment before and during the pandemic was significant or not while the number of missed appointment was used as outcome variable when we assess the determinants of missed appointment. In our case, the observations are not independent and then it is possible to consider paired t-test to determine the difference in proportion of missed appointments before and during the pandemic. However, we only estimate the proportion missed appointment before and during the pandemic with its 95%CI.

3. In the Results, the authors described how they landed on negative binomial regression because of over-dispersion. I recommend a) In Methods, make clear that number of missed appointment is the dependent variable in the negative binomial regression; b) consider the dependence of observations in the regression; c) consider including number of scheduled appointments as an offset in the regression model (natural log(number of scheduled appointments)); when the offset is used, they should remove “The more frequent the visits, the higher the incidence of missed appointments” in page 14, and should not include duration of follow-up because number of scheduled appointments already contained all the information needed.

**Authors’ response:** Thank you dear reviewer for your concern. We revise according to your comments. Yes, it is better to consider offset variable in count data analysis. Despite the number of missed appointment, duration of follow up is an offset variable for us Because an individuals with longer duration of variable had higher risk of missed appoint and vice versa and. Regarding to the independent assumption, we only use the frequency of missed appointment as dependant variable and consider missed appointment history before he pandemic as predictor. Therefore, independent assumption is not an issue for us.

4. It was not clear what criteria were used to determine that negative binomial regression model was the best. Please provide a reference in page 14.
   **Authors’ response:** Thank you dear reviewer for your concern. The commonly used count data analysis is Poisson distribution with constrained assumption which is the mean and variance should be equal. Because of the violation of this assumption, we consider negative binomial regression.
5. These sentences in Discussion are confusing: “Still, after a year, the pandemic remains very challenging, especially for low-income countries. There have been different strategies and non-pharmaceutical interventions implemented, however, the implementation level has been very limited due to the cultural and traditional practices in the country.” We are in the third year of the pandemic. I do not know what “strategies” and “interventions” were referred to and for what purposes. Suggest removing these sentences.

**Authors’ response:** Thank you dear reviewer for your concern. Yes we are in the third year but this study was based on the data a year after the declaration of pandemic. We have revised it in the main document.

6. The author did not distinguish missed appointments, cancelled appointments, and rescheduled appointments. Were cancelled and rescheduled appointments counted as missed appointments? If they were, how many of those missed appointments were cancelled or rescheduled? Please discuss this issue.

**Authors’ response:** Thank you dear reviewer for your concern. We consider both cancelled and rescheduled appointments counted as missed appointments. But we consider it as our limitation because considering rescheduled appointments as missed appointments might overestimate the number missed appointments.

7. There were many grammar-like errors in the manuscript, some examples are: a) lower case for “No-show rate” in page 5; b) there should be a space between 1005 and (55.37%) in page 8; c) throughout the manuscript, insert a space in 95%CI (like 95% CI); d) need define abbreviations before using them (e.g., CIRR, AIRR); e) decimals were not consistence, for example, in Table 3, column 4, some numbers had two decimals and other had three decimals; also in the Table 3, some had no space between a number and left parathesis. f) it seems that the last sentence of the Results is not complete: “Nearly 40% increase in …..”. g) in page 17, “The possible reason may be because patients may fear contracting the pandemic if…” should be “The possible reason may be because patients may fear contracting the novel coronavirus if…”; h) in page 17, “experienced COVID-19 like symptom(s) sedentary lifestyle were found to ...” should be “experienced COVID-19 like symptom(s), and sedentary lifestyle were found to …”

**Authors’ response:** Thank you dear reviewer for your concern. We tried to revise the overall manuscript regarding to any typological and grammar errors.
