# Supplementary material for: Effect of COVID-19 pandemic on missed medical appointment among adults with chronic disease conditions in Northwest Ethiopia
Source: PLoS One. 2022 Oct 4;17(10):e0274190. doi: 10.1371/journal.pone.0274190 (PMC9531804; doi:10.1371/journal.pone.0274190)
Supplement: Attachment — Submitted filename: Response to Reviewers-R2.docx [file pone.0274190.s004.docx]

August, 2022

**Rebuttal letter**

**Manuscript ID:** PONE-D-21-28858R1

**Title:** Effect of COVID-19 Pandemic on Missed Medical Appointment among Adults with Chronic Disease Conditions in Northwest Ethiopia

*Tadesse Awoke Ayele, Tesfa Sewunet Alamneh, Habtewold Shibru, Malede Mequanent Sisay, Tesfahun MeleseYilma, MelkituFentie Melak, Telake Azale Bisetegn, Tariku Belachew, Mahteme Haile, Taye Zeru,Mezgebu SelamsewAsres, and Kegnie Shitu*

**Dear Editor,**

We would like to thank for your consideration and suggestion for the betterment of our manuscript and make it more informative. We tried to amend the format of the manuscript according to the journal guidelines and address the questions/suggestions forwarded by the editor. Our point-by-point responses for each comment and questions are described in detail on the following pages. Further, the details of changes were shown by track changes in the supplementary document attached to the online submission system. Thanks.

**Editor’s comment**

Appreciate that a conclusion was added, but it was too long, suggest the following:

The rate of missed appointment increased significantly during the COVID-19 pandemic. Older age, longer duration of follow up, more frequent follow-up, out-of-pocket expenditure for health service, history of poor follow-up, and sedentary lifestyle had positive relationship with missed appointments during the pandemic. Therefore, it is important to give special emphasis to individuals with these risk factors while designing and implementing policies and strategies for peoples with chronic diseases to ensure the continuity of care and to avoid the long-term impact on their health.

**Response:** Thank you so much for the comment and suggestions: The revision has been made accordingly (**See the conclusion in the abstract of the revised manuscript**).

Need add the following before the last sentence of "Data Collection Methods and Measurements"

We extracted two-year’s data from medical chart (the primary data source). For those variables that were not recorded in the chart, we conducted patient interview to collect the data (the secondary data source).

**Response:** Thank you. Changes have been made accordingly (**See the revised manuscript line 146-147 on page 7 of the revised manuscript**)

It is good to see reasons for strengths and limitations. But there are some language issues. Suggest changing

“The other limitations include retrospective nature of the study might underestimate the outcome variable. Because when the date at for an appointments were not documented, we could not assured weather an individual missed the appointment or not. Moreover, this study considers both cancelled and rescheduled appointments as missed appointments. Though considering rescheduled appointments as missed appointments might overestimate the number missed appointments.”

to

“Another limitation is the retrospective nature of the study. When the date for an appointment was not documented, we could not determine whether an individual actually missed the appointment or not. Moreover, this study considered both cancelled and rescheduled appointments as missed appointments and might have overestimated the number of missed appointments.”

**Response:** Revision has been made accordingly and your suggestions were super helpful (**See the revised manuscript line 379 to 383 on page 21 of the revised manuscripts**).

**Journal Requirements:**

Please review your reference list to ensure that it is complete and correct. If you have cited papers that have been retracted, please include the rationale for doing so in the manuscript text, or remove these references and replace them with relevant current references. Any changes to the reference list should be mentioned in the rebuttal letter that accompanies your revised manuscript. If you need to cite a retracted article, indicate the article’s retracted status in the References list and also include a citation and full reference for the retraction notice.

**Response:** References have been double checked. We couldn’t not any retracted article in our reference list. However, we have made some revisions on some of the references involving adding URL address for internet/website references (**See the reference section of the revised manuscript with track changes**).
